# Functional Tricuspid Regurgitation: Behind the Scenes of a Long-Time Neglected Disease

**DOI:** 10.3389/fcvm.2022.836441

**Published:** 2022-02-21

**Authors:** Mattia Vinciguerra, Marta Sitges, Jose Luis Pomar, Silvia Romiti, Blanca Domenech-Ximenos, Mizar D'Abramo, Eleonora Wretschko, Fabio Miraldi, Ernesto Greco

**Affiliations:** ^1^Department of Clinical, Internal Medicine, Anesthesiology and Cardiovascular Sciences, Sapienza University of Rome, Rome, Italy; ^2^Cardiovascular Institute, Hospital Clinic, University of Barcelona, Barcelona, Spain; ^3^Institut D'Investigacions Biomèdiques August Pi i Sunyer (IDIBAPS), Barcelona, Spain; ^4^Centro de Investigación Biomédica en Red (CIBERCV), Instituto de Salud Carlos III, Madrid, Spain; ^5^Department of Cardiac Surgery, Clinic Barcelona Hospital University, Barcelona, Spain; ^6^Department of Cardiac Surgery, Barnaclinic, Barcelona, Spain; ^7^Department of Radiology, Hospital Clínic, Barcelona, Spain

**Keywords:** functional tricuspid regurgitation, right ventricle, annuloplasty, transcatheter approach, prosthetic ring

## Abstract

Severe tricuspid valve regurgitation has been for a long time a neglected valve disease, which has only recently attracted an increasing interest due to the notable negative impact on the prognosis of patients with cardiovascular disease. It is estimated that around 90% of tricuspid regurgitation is diagnosed as “functional” and mostly secondary to a primary left-sided heart disease and, therefore, has been usually interpreted as a benign condition that did not require a surgical management. Nevertheless, the persistence of severe tricuspid regurgitation after left-sided surgical correction of a valve disease, particularly mitral valve surgery, has been associated to adverse outcomes, worsening of the quality of life, and a significant increase in mortality rate. Similar results have been found when the impact of isolated severe tricuspid regurgitation has been studied. Current knowledge is shifting the “functional” categorization toward a more complex and detailed pathophysiological classification, identifying various phenotypes with completely different etiology, natural history and, potentially, an invasive management. The aim of this review is to offer a comprehensive guide for clinicians and surgeons with a systematic description of “functional” tricuspid regurgitation subtypes, an analysis centered on the effectiveness of existing surgical techniques and a focus on the emergent percutaneous procedures. This latter may be an attractive alternative to a standard surgical approach in patients with high-operative risk or isolated tricuspid regurgitation.

## Introduction

Right ventricle (RV) overload and tricuspid annular (TA) dilatation have been identified from the outset as the main causes in the pathogenesis of tricuspid regurgitation (TR) following left heart valvular disease (LHVD). The absence of primary abnormalities of the valvular apparatus has led TR, from the earliest studies, to be approached as of a “functional” nature mediated by pressure and volume overload that affect the geometry of RV, right atrium (RA), and TA (1, 2). In a simplistic manner, an inadequate systolic closure in the presence of a structurally normal valvular apparatus may define the functional TR (FTR). The complexity emerges when leaflet coaptation is assessed in the setting of the disease, with different etiological mechanisms, besides LHVD, responsible in triggering annular and/or subvalvular morphological modifications (3). The growing interest for TR and the advances in imaging, three-dimensional (3D) echocardiography and cardiovascular MR (CMR) among others, have allowed FTR to be categorized in more detail. The pathophysiological comprehension of the different TR phenotypes, identified according to the etiological mechanism and the associated natural history, may significantly help clinicians and surgeons in the decision-making.

Indeed, in the setting of LHVD, both the severe mitral regurgitation and aortic stenosis have a high percentage, up to 50 and to 25%, respectively, and the concomitant presence of at least moderate TR, with worsening or late onset of the TR when the solely left heart valvular disease was surgically corrected (4–11).

In the light of these findings, the guidelines, supported by the absence of an increased operative risk, recommend tricuspid valve (TV) surgery during the surgical management of left-sided disease, with an improvement in RV function and functional status, if the disease is not very advanced (12).

In contrast, knowledge about the natural history and the timing of intervention in isolated TR remain poor because its clinical onset usually occurs in an advanced stage of the disease, which may increase periprocedural morbidity and mortality (12). Moreover, there is also scarce evidence on the role and timing of TV surgery and newer transcatheter repair approaches.

## Pathogenesis Of FTR

In the pathogenesis of FTR, valvular coaptation results impaired due to mainly two mechanisms: TA dilatation with distortion of its morphology and restricted motion of leaflets caused by increased tethering.

Due to the peculiar anatomy of TV, when the functional dilatation of TA occurs, typically, the septal portion is spared, involving the anterior and the posterior leaflet attachment. Thus, the normal “saddle-shaped” structure is altered toward more circular and planar geometry (3).

The magnitude of annular dilatation correlates with greater degrees of TR and, in particular, the threshold beyond which coaptation results significantly inadequate is notably lower than for mitral regurgitation, with 40 rather than 75%, respectively (13, 14).

Therefore, the loss of coaptation is importantly influenced by the dilatation and distortion of TA, often coexisting with leaflets pulled down the annular plane due to increased tethering. In this latter case, anterior and posterior papillary muscles (PMs) are displaced due to RV cavity change in size and shape (15).

An *in-vitro* study that simulated annular dilatation and PMs displacement showed that when considered alone, both the conditions may lead to significant FTR (14). This finding highlights the importance of carefully studying the primary disease processes that cause FTR.

According to the recent literature, FTR may be classified as secondary to:

- Left heart valvular disease.
- Right heart dysfunction (RHD) with or without pulmonary hypertension (PH).
- Right atrium enlargement and dysfunction.

In the majority of FTR cases, the primary triggering condition is LHVD, mainly chronic mitral regurgitation followed by aortic valve stenosis and mitral valve stenosis (16, 17).

The different causes of FTR determine significant anatomic differences in RV, TV, and TA anatomy.

Interestingly, FTR secondary to PH, mainly associated to LHVD or in the setting of primary PH due to pulmonary disease such as chronic pulmonary embolism among others, showed only mild annular dilatation, but excessive valve tenting height with a conical deformation of RV (16).

The progressive RV dilatation, promoted by the relative lack in muscular tissue of RV, due to the increased afterload, is triggered by PH. Different studies have demonstrated the role of PH as a determinant of FTR severity; in a vicious circle, the chronic pressure and volume overload increase leaflets tethering, ultimately worsening TR (18).

Besides the absolute value of pulmonary artery systolic pressure in defining the severity of the PH, the onset of significant FTR is strongly associated with right heart remodeling (19). Indeed, in the absence of PH, RV dysfunction due to ischemic injury or cardiomyopathy may lead to adverse remodeling, PMs displacement, and increased leaflets tethering, causing FTR (20).

On the other side of the spectrum lesions, FTR with a larger basal deformation of the RV and a lower annular/leaflet coverage ratio due to greater annular dilatation, typically occurs due to RA enlargement and dysfunction (16).

Atrial FTR (AFTR) was for long time called idiopathic FTR, so categorized due to the absence of any obvious cause; recently, its accurate study has allowed to revise the pathophysiological classification, awarding to the RA a primary role as its main determinant (17, 21–23).

Indeed, current literature has emphasized the underappreciated issue associated to the atrial failure and its prognostic implication in worsening natural history of heart failure among others (24).

Atrial FTR reflects the TV leaflets malcoaptation because of the imbalance between TA and leaflets area, caused by RA enlargement and dysfunction.

The advanced age and the presence of persistent/permanent atrial fibrillation (AF) are typically associated to the AFTR, reflecting as common denominator of the atrial contractile dysfunction (21, 23). In particular, the onset of AF may modulate myocytes action potential and calcium release from the sarcoplasmic reticulum; regardless by AF, in the elderly, the frequent diastolic dysfunction may lead to an increase in atrial pressure and atrial stretch (25, 26).

In the study of Utsunomyia et al. (27), who compared subjects affected by AFTR and ventricular FTR, the assessment of the RA remodeling has allowed to conclude that RA volume and its ratio with RV end-systolic volume are independent predictor of TA volume in AFTR.

Interestingly, they stratified AFTR according to severity of regurgitation in severe and massive/torrential TR, showing in this latter more complex lesions with excessive TA dilation associated to different degrees of leaflets tethering (27).

What emerges by the afore discussed categorization is a strong association between RV, RA, and valvular changes and in the light of this complex interaction, FTR may be interpreted as a complex syndrome with multifaceted spectrum of pathogenesis, natural history, and outcome.

Therefore, the qualitative evaluation of FTR based on pathophysiological categorization should be integrated with quantitative measurements and parameters exploited by imaging to improve the decision-making process and offer individualized management for our patients.

## Diagnostics

### Echocardiography

The first-line imaging recommended in the assessment of TR is the transthoracic echocardiography, which is useful to provide information regarding to the main determinants of the disease and to estimate its severity.

In the setting of FTR, the assessment of TV anatomy, in order to exclude any structural abnormalities, anticipates the measurement of TA diameter, RV size and function, RA size, and the estimation of pulmonary artery pressures (PAPs) (28).

The measurement of TA diameter has represented the critical component univocally used to guide the procedural planning of TV intervention. The cutoff value of 40 mm or 21 mm/m^2^ indexed for the body surface area has been established and still used as threshold in surgical decision-making to approach TV in concomitant procedures. Nevertheless, the two-dimensional (2D) echocardiographic measurements fail to consider the 3D nonplanar structure of TA and in addition the interindividual variability. In comparison, 3D echocardiography, obtained by the transthoracic window rather than the transesophageal one because of the better visualization, is significantly less influenced by TA shape and orientation (28).

The comprehensive study of RV size and function has notably implications in the accurate categorization of FTR and it has an important prognostic value (29). Indeed, the geometrical modification of the right chamber may be influenced by primary abnormalities in the setting of RHD and indirectly due to LHVD, leading to elliptical/spherical shape and increased leaflets tethering.

The evaluation of RV size and function is a key determinant of prognosis and potential intervention in patients with TR. RV evaluation with echocardiography is challenging due to the complex 3D geometry of the RV. Typically, the first approach to the RV includes RV size estimation from the four-chamber view, evaluating its diameter at the basal level (normal up to 42 mm) and also its relative size as compared to the left ventricle (LV) (the normal RV is always smaller than the LV). Qualitative estimation of RV systolic motion from this view also provides a first impression of RV systolic motion. Longitudinal shortening of the basal segments is the main contributor to global RV function; therefore, the assessment of global function can also be provided by measuring the tricuspid annular plane systolic excursion (TAPSE) from M-mode scans through the TA in the four-chamber view (abnormal below 17 mm). Determination of peak systolic annular velocity (S wave from tissue Doppler imaging of the tricuspid annulus) also provides a global index of RV systolic function (values below 11 cm/s indicate systolic dysfunction). However, both the TAPSE and annular S wave values are influenced by loading conditions. Volumetric methods to assess RV ejection fraction are cumbersome by using 2D echocardiography due to the particular geometry of the RV and, therefore, 3D echocardiography, despite limited by visual quality and spatial resolution, is preferred. In the absence of 3D echocardiography, RV fractional area change obtained from the planimetry of the endocardial border of the RV performed in the four-chamber view and its relative change in systole and diastole is typically used to assess RV systolic function. Finally, myocardial deformation imaging determining global longitudinal strain of the RV is another essential tool in the evaluation of RV systolic function that can be successfully obtained with echocardiography. Typically, the free wall RV global strain is obtained from the four-chamber view with normal values above 20% or even higher in the presence of volume overload such as the case of patients with TR. This parameter has shown added prognostic value in several clinical settings such as cardiogenic shock and PH (30, 31).

The extent of leaflet tethering (including the measurement of tethering distance, area, and volume), the mode of leaflet coaptation, and the RA size offer supplementary information regarding the natural history of the FTR, allowing to stage the severity of the disease according to the complexity of lesions (28).

**Figure 1:**
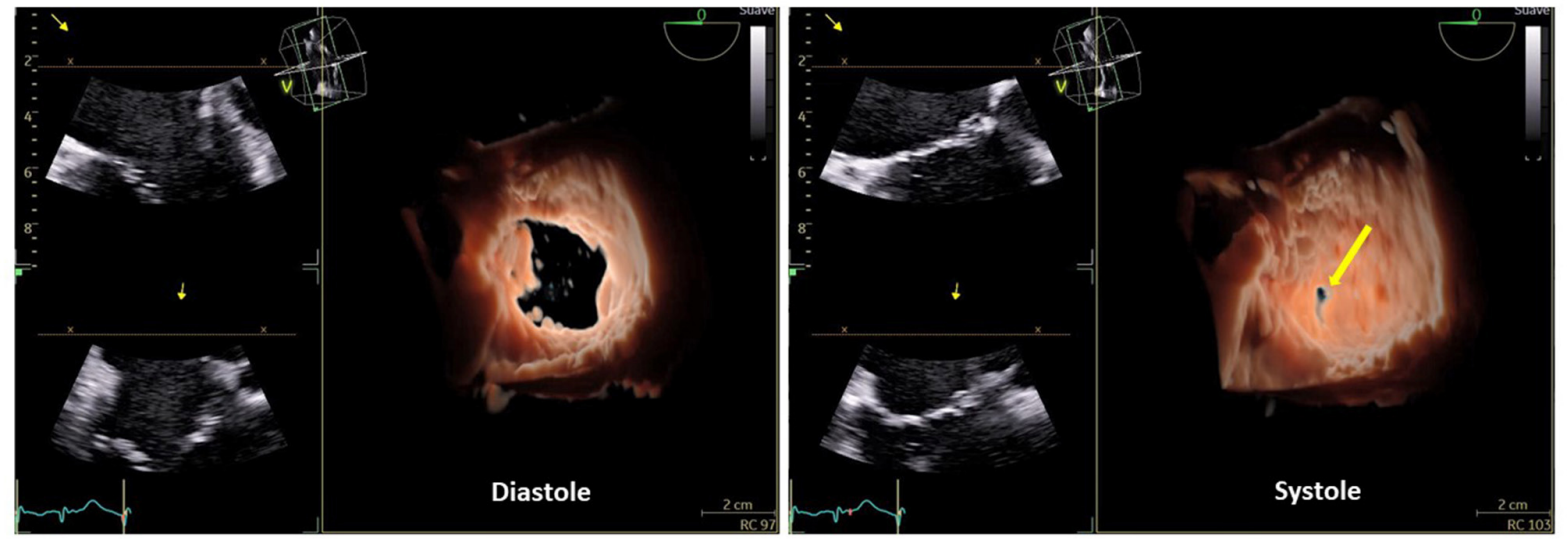
Three-dimensional (3D) echocardiography of the tricuspid valve with front view, from the right atrium in diastole and systole where the central coaptation defect (yellow arrow), due to annular dilation, is seen.

Besides the qualitative description improved by the use of 3D echocardiography (Figure 1), the severity of TR needs to be related to quantitative parameters, in order to make reproducible its staging. The measurement of the diameter of the vena contracta of the regurgitant jet and the tricuspid effective regurgitant orifice obtained by the proximal isovelocity surface area (PISA) method are the largely used parameters. The cutoff used to estimate the severity of TR is similar to those identified for mitral valve regurgitation, with 0.70 cm and 0.40 cm^2^, respectively, for the vena contracta and for the effective regurgitant orifice area, as threshold to quantify severe TR.

Several issues affect the staging role assigned to these quantitative values; in particular: the anatomical difference existing between TV and its left counterpart, associated to the higher respiratory function and loading variability. The 3D echocardiography allows to partly overcome the anatomical disagreement, even though failing to solve the limitations related to the assessment of a single frame developed on a tomographic plane in the setting of a variable dynamic disease (28, 32).

### Cardiac Magnetic Resonance

**Figure 2:**
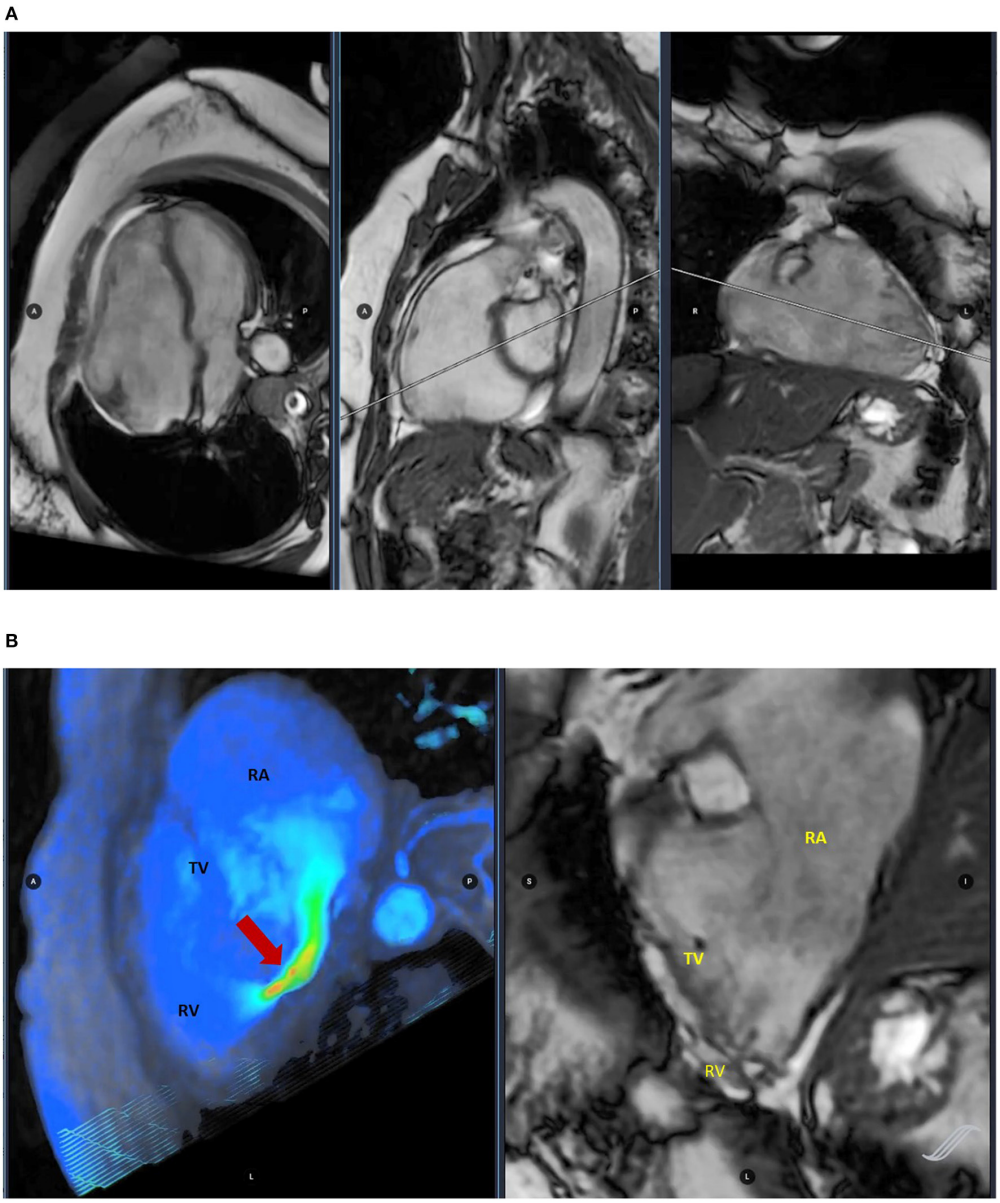
Cardiac magnetic resonance (CMR) imaging assessing ventricular volumes **(A)** and four-dimensional (4D) flow image evaluating tricuspid regurgitation (TR) (red arrow) **(B)**.

Patients with TR are a heterogeneous population at different stages of right heart remodeling. CMR is the gold standard imaging technique for the quantitative and qualitative assessment of the heart, particularly the RV morphology and function and, indeed, it has diagnostic value and it can provide valuable information to improve the decision-making process on the management of TR (Figures 2A,B) (33).

Cardiac magnetic resonance allows a comprehensive assessment of FTR, starting from the qualitative visualization of valve regurgitation with flow turbulence and acceleration visualized in the cine images as an area of local signal drop. The qualitative grading has shown a moderate correlation with the quantitative assessment, although influenced by different limitations such as the difficult arising from the correct visualization in presence of pacemaker leads and the bias associated to various technical factors.

The more accurate quantitative measurement, although limited by existing validated cutoff and, therefore, the still poor experience, allows to calculate the regurgitant volume (RVol) through TV with both the indirect and direct methods. In the first case, RVol is derived from the right ventricular volumetric stroke volume, demonstrating to be a good prognostic predictor in patients underwent to the surgical management of isolated TR.

In contrast, the direct calculation of RVol depends by the flow assessment through the valve, but it is still challenging due to intrinsic factor associated to the atrioventricular annuli (34).

Moreover, the determination of the RV morphology and RV cavity size assessed by CMR cine-sequence images has been proved to have prognostic value in different clinical settings (35, 36). Recently, Iacuzo et al. (37) have highlighted the fundamental role that TA plays in the surgical decision to perform preventive combined TV annuloplasty in patients with mitral valve prolapse. They found that the best cutoff for the prediction of RV dysfunction in these patients is a TA diameter index value of 19 mm/m^2^ measured by CMR, beyond which combined TV annuloplasty should be considered.

Besides heart cavities assessment, CMR can also accurately evaluate the pulmonary vasculature and it has been proposed as an alternative to invasive right heart catheterization and echocardiography in assessing pulmonary circulation hemodynamics and to estimate pulmonary vascular resistance (6). The CMR-derived model was estimated as: pulmonary vascular resistance [in Wood units (WUs)] = 19.38 – [4.62 × Ln pulmonary artery average velocity (in cm/s)] – [0.08 × RV ejection fraction (in %)] (24). This CMR method to noninvasively estimate pulmonary vascular resistance has been used in patients with PH and also in patients with heart failure and reduced LV ejection fraction (EF), in whom pulmonary vascular resistance > 5 WU was associated with an increased risk of adverse events at 9 months follow-up (38, 39). Based on all this, we believe that this CMR method could also be integrated in the global evaluation of the RV in the setting of FTR in order to better understand if the RV contractile reserve is struggling to maintain output against an increased afterload, a finding which, indeed, previous studies have shown that entails a poor prognosis (39).

Additionally, myocardial strain has received growing interest as an alternative measure of myocardial performance since it has proven to better recognize and characterize subclinical biventricular systolic dysfunction as compared with conventional imaging modalities based on EF (40, 41). In fact, it is well known that impairment in the longitudinal deformation precedes the reduction in EF, giving rise to subclinical pump impairment in most heart diseases (42, 43). CMR feature tracking (FT) allows the assessment of strain from routine cine images and besides its diagnostic value, it might also have a prognostic value in patients with TR. Recently, Romano et al. (44) found that RV longitudinal strain assessed by CMR-FT is an independent predictor of mortality in patients with severe FTR, incremental to common clinical and imaging risk factors.

Better identification of high-risk patients that benefit from a closer follow-up together with the implementation of a global RV imaging evaluation that includes the assessment of estimated PVR and the early detection of RV dysfunction by using FT may potentially play a role in determining optimal timing of intervention of patients with FTR.

## Therapy

A recent growing surgical interest in the management of FTR has reversed course, polarized in the past by the wrong concept of disease regression after properly solving LHVD (1).

The consequence of the less attention received rather than for mitral valve regurgitation has been easily translated in a still lacking surgical experience in offering a targeted approach.

Although, in absence of a clear evidence demonstrating superiority of one surgical approach over others, TV repair, mainly annuloplasty, represents the preferred options in the management of FTR. A growing interest emerged at the end of 90' in various minimally invasive and video-assisted surgical approaches (45–50). TV replacement may be a viable option when risk for TR recurrence after repair is considered too high.

A great challenge, in the surgical decision-making, is represented by the management of electrical conduction abnormalities in concomitance with TR, which can facilitate the need for pacemaker implantation in the postoperative period. An accurate evaluation of the possibility to implant epicardial leads is necessary in order to reduce the risk of TR recurrence mainly due to prosthesis leaflets damage following the traditional leads implantation.

In this paragraph, we review the most commonly TV repair techniques to offer a focus insight into the future perspectives of surgery in the management of FTR in the era of transcatheter approach. Tables 1, 2 summarize, respectively, the main characteristics of the principal FTR surgical techniques and the transcatheter devices.

**Table 1:** Summary table with the description of the principal surgical technique in the correction of the functional tricuspid regurgitation (FTR).

**Type of technique/device**	**Description**	**Strength**	**Limitation**
Suture bicuspidization	Double pledget-supported mattress suture from the anteroposterior to the posteroseptal commissure	Easily reproducible	frequent technical failures
De Vega annuloplasty	Double continuous running suture along anterior and posterior portions of the annulus	Safe, effective, easily reproducible, cheap	Occasional Suture tear: “Bowstring” phenomenon
Prosthetic annuloplasty: - Duran-Medtronic flexible ring - Carpentier-Edwards semi-rigid ring - Cosgrove-Edwards flexible band - Duran-Medtronic flexible band and others …	Implantation of a prosthetic band or ring	Reduction of recurrent dilatation by a non expandable frame	May Need additional gestures in complex lesions (excessive leaflets tethering)
Papillary muscles septalization	The approximation of the anterior PM, attached to the RV free wall and more prone to displacement, toward the interventricular septum	The technique allows to increase the surface of coaptation of leaflets reducing the rate of TR recurrence	Lack of large series reported
Leaflet augmentation	Leaflet augmentation using a pericardial patch	The technique allows to increase the leaflets surface improving systolic area closure	Lack of large series reported Eventual degeneration of pericardial patch (stiffer and retraction)

**Table 2:** Summary table with the description of the more used transcatheter technique/device of tricuspid valve intervention.

**Type of technique/device**	**Description**	**Strength**	**Limitation**	**Ongoing trials**
**Transcatheter tricuspid valve replacement**				
Transcatheter tricuspid valve replacement: -Orthotopic implantation -Heterotopic implantation	Implantation of a prosthetic valve in the tricuspid location or in the vena cava	Alternative option to repair techniques, mainly in patients with degeneration of previous tricuspid valve correction	Lack of experience	
**Leaflets coaptation device**				
TriClip (Abbott, Chicago, Illinois)	Edge-to-edge repair	Satisfactory reduction of tricuspid regurgitation and improvement in the functional class for patients not suitable for surgery	Lack of comprehensive data on eligible patients	
FORMA system (Edwards Lifesciences, Irvine, California)	Implantation of a balloon spacer anchored to right ventricle apex able to reduce regurgitant orifice area	Alternative options to edge-to-edge repair	Invasiveness of the device	
**Suture annuloplasty devices**				
- Trialign (Mitralign Inc, Tewksbury, MA) - Tricinch (4Tech Cardio Ltd., Galway, Ireland)	System of anchors placed on the anterior and posterior segments of the tricuspid annulus	Reduction of tricuspid annulus diameter in patients not suitable for surgery	Lack of mid-term follow-up data	Safety and Feasibility of the Transcatheter Tricuspid Valve Repair System (Trialign) **Trials Identifier:** NCT04936802
**Prosthetic annuloplasty devices**				
- Cardioband system (Edwards Lifesciences, Irvine, CA, USA) - Millipede IRIS (Millipede Inc., Santa Rosa, CA, USA)	Transcatheter implantation of a prosthetic annulus	Reduction of tricuspid regurgitation in patients deemed inoperable	Lack of mid-term follow-up data	

### Tricuspid Valve Annuloplasty Techniques and Outcomes

The cornerstone of the TV repair techniques has found its foundation in counteracting the dilatation of TA, with annuloplasty being the most widely used approach.

First, the way of the annular plication was undertaken following the concept of the suture bicuspidization. The posterior annular portion of TV, being the less unsupported segment of TV ring, dilates achieving up to 80% from its normal dimension in the setting of FTR. This anatomic finding was at the basis of suture bicuspidization technique (51). The technique, initially described by Kay et al. (52) and then modified by Ghanta et al. (53), consists in placing a double pledget-supported mattress suture from the anteroposterior to the posteroseptal commissure, resulting in a posterior suture bicuspidization.

Although highly reproducible, the tension created by the suture lines and the high technical failure associated to severe TA dilatation have limited the use of the suture annuloplasty toward alternative strategies such as the implantation of prosthetic ring or band (54).

De Vega proposed, after understanding the way TA was dilated, a double continuous running suture with the aim to plicate the anterior and the posterior portions of the annulus avoiding the septal due to the lack of distension and to avoid the conduction tissue. Even though this semicircular suture annuloplasty was initially designed for rheumatic TV disease, it reached a great success in the management of FTR, mainly because it was safe, effective, at least in the short term, easily reproducible, and cheap (54, 55). Nevertheless, even if a series of De Vega annuloplasty modifications has been developed in order to avoid suture tear and massive TR, this phenomenon, called “bowstring,” was rather frequently reported after suture annuloplasty (54).

The technical aspects of the various De Vega annuloplasty modifications followed the rationale to achieve better coaptation, reduced annular dimensions, and a better distribution of forces along the suture in order to prevent tear in the endocardium (56–60). The absence of constant mid- and long-term follow-up has limited a wider reproducibility of some of these techniques.

Alain Carpentier, emphasizing the criticisms associated to the principles of the suture annuloplasty, proposed the use of suitably sized and shaped prosthetic rings. In the former, the asymmetric placement of the sutures results in localized plication and in variable degrees of functional stenosis. On the contrary, the rationale for the implantation of a prosthetic rigid ring was based on the respect of anatomic and physiologic TA geometry, allowing to avoid recurrent dilatation with a nondeformable frame (61).

The Duran-Medtronic totally flexible tricuspid and the Carpentier-Edwards semi-rigid rings were later followed by the flexible Cosgrove-Edwards and Duran-Medtronic bands.

The flexible band was specifically designed on the basis of the improved knowledge regarding dynamic saddle-shaped structure of TA and about its pathological dilatation that involves particularly anterior and posterior segments (62).

McCarthy et al. (63) compared the durability of tricuspid annuloplasty in the management of FTR by using four techniques: De Vega procedure, Peri-Guard annuloplasty, Carpentier-Edwards semi-rigid ring, and Cosgrove-Edwards flexible band. In the long-term follow-up, a substantial worsening in the recurrence of TR was associated to the two nonring annuloplasties. Indeed, along the follow-up, severity of regurgitation increased more rapidly when these procedures were performed, slower with the flexible band and more stable across time with semi-rigid ring. The authors explained the higher recurrence of severe FTR in the gradual redilatation of the TA due to the persistent PH, in the absence of a nondeformable frame.

The superiority of the prosthetic annuloplasty ring, in ensuring a higher durability of FTR correction, a better long-term survival, and event free-survival, was largely demonstrated (64–68).

Nevertheless, the main finding of the retrospective study published by McCarthy et al. (63) was the failure of the tricuspid valve annuloplasty, regardless by the implantation of a prosthetic ring, to consistently counteract functional regurgitation. Even though stable across time, the prevalence of severe regurgitation 1 month postoperatively was 15% for the semi-rigid ring technique. This latter allowed to achieve higher freedom from moderate-to-severe TR when compared with flexible bands (69).

Over the years, a variety of prosthesis for annuloplasty has been presented, notably ensuring improvement in remodeling TV annulus, respecting the 3D anatomical dynamic characteristics. The development of the 3D-shaped prosthetic rings has allowed to resemble more accurately the healthy human TA, reducing the recurrence of the TR in the long-term follow-up, with an incidence reported in a recent study, which compared the use of the Medtronic 3D Contour and the Edwards MC3 prosthetic rings, of almost 15% of TR > 2+, mainly of moderate nature (70, 71). Newer rings are today commercially available, but their long-term outcomes are still to be reported in the scientific literature.

### Current and Future Perspectives

#### “Prophylactic” Annuloplasty

The late onset of TR after left-sided surgery, regardless by the presence of preoperative TR, highlights the dynamic nature of TR, which, in contrast with what Nina Braunwald believed, does not regress spontaneously (1). Dreyfus et al. (7) used the annular dimension as target to address TV, including patients only with none or mild trace of TR. The chosen threshold to manage TV, in the setting of left-sided surgery, was a diameter greater than twice the normal size (i.e., > 70 mm) inspected intraoperatively. The cutoff allowed to divided population study in the two groups of treatment: mitral valve repair (MVRe) alone and MVRe plus tricuspid annuloplasty. This latter group showed better outcomes in terms of late recurrence of TR; postoperative TR grade increased significantly in the group underwent isolated MV surgery (7). These results demonstrated the irreversible progression of annular dilatation, being not influenced by the correction of the LVHD, and introduced the concept of the “prophylactic annuloplasty”.

The echocardiographic equivalent of the intraoperative threshold has been identified in 40 mm. Tricuspid annuloplasty was performed, in addition to left-sided surgery, in patients with TA dilatation more than 40 mm, independently by the grade of TR, in a cohort of 43 patients in the study published by Van de Veire et al. (6).

The authors analyzed retrospectively two cohorts temporally distinct, with the introduction of the “prophylactic annuloplasty” in the management of FTR in the more recent group of patients. A significant decrease of TR, transtricuspid gradient and a reduction of the RV volumes leading to reverse remodeling were observed (6). These findings have been confirmed by other studies (72, 73). The satisfactory outcomes achieved and the low in-hospital mortality and morbidity associated to the TV annuloplasty, although increasing operation time, promoted this preventive treatment to become recommended, according to the valvular heart disease guidelines, during left-sided surgery (74, 75).

Benedetto et al. (72) observed, at 12 months follow-up with preoperative TR ≤ 2+, no cases of moderate-to-severe TR in the group, which underwent tricuspid annuloplasty vs. 28% in the control group (isolated MV surgery).

Interestingly, the subgroup analysis demonstrated an advantage of TV annuloplasty toward TR caused by rheumatic or ischemic injury, failing to show significant benefits in degenerative etiology. The authors explained the ineffectiveness of the additional treatment identifying some predisposing risk factors such as worse functional class and the presence of AF, responsible of long-standing RV pressure overload.

In addition, the high variability correlated to the broad range of degenerative disease raised various questions about the predictivity of TA diameter as a valid cutoff for decision-making (76).

Although tricuspid annuloplasty represents a coherent surgical approach in counteracting TA dilatation in the early stage of disease when LVHD correction is performed, a single threshold in the setting of a multifaceted syndrome is a limitation in achieving excellent results. Studies, tailored on the different etiology and pathophysiology, designed in order to identify targeted parameters, are mandatory.

#### Valvular and Subvalvular Repair Techniques

The wrong assumption that TA dilation is always the primary cause of leaflet malcoaptation and that TV annuloplasty is able to correct FTR counteracting also leaflet tethering, is generally incorrect; in fact, there is a high early residual rate of TR when tethering is excessive (76). Indeed, Fukuda et al. (77) have demonstrated the role of TV tethering in predicting residual TR early after surgery; in particular, identifying as significant values: tethering height > 0.76 cm and tethering areas > 1.63 cm^2^.

Therefore, the geometry of the TV can vary according to the major determinants of its severity, TA dilatation, leaflet tethering (particularly of the septal leaflet), and the grade of PH, designing a complex variety of lesions (78).

In such cases, additional surgical strategies in order to improve results of TV repair are necessary, allowing to reduce early technical failure and TR recurrence.

Surgery at subvalvular level with PMs approximation has demonstrated to be effective in reducing TR independently by the use of tricuspid annuloplasty and it is able to promote changes in the RV geometry leading to reverse remodeling. The technical aspects of this surgical strategy are based on the approximation of the anterior PM, attached to the RV free wall and more prone to displacement, toward the interventricular septum (79).

Excellent outcomes have been achieved by using PMs “septalization” in the management of the massive FTR caused by distorted geometry of the TV and adverse remodeling of the RV (80–83).

In the setting of severe tethering, the restricted mobility leads to the failure of the leaflets in covering the whole orifice; leaflet augmentation using an autologous pericardial patch found its rationale in this concept. Dreyfus et al. (84) promoted to use this technique when annuloplasty ring alone is not adequate to impact significantly on the TR when a tethering height <8 mm has been assessed during preoperative echocardiography. The increase of the surface area of coaptation, combined with TV annuloplasty allows to counteract FTR caused by complex lesions, accomplishing excellent short-term results (85, 86). Stiffness of the pericardial patch and retraction have been mentioned as potential long-term limitations.

#### Transcatheter Approaches

The great technological improvements and the experience achieved in the field of the transcatheter repair or replacement of the aortic and mitral valves have accelerated the use of the transcatheter tricuspid valve interventions (TTVIs) in high-risk surgical patients. The possibility to recur to a less invasive surgical option of treatment, in particular in selected patients such as who underwent to previous TV surgery, allows to reduce the high incidence of postoperative mortality and morbidity associated to the reintervention. Nevertheless, the lack of a complete understanding of the role and timing of the TV surgery represented a limitation in allowing a standardized management (87).

A broad armamentarium including multiple devices aimed to repair and replacement has been developed in the field of tricuspid disease.

Transcatheter TV replacement includes the orthotopic implantation of a prosthetic valve in the tricuspid location or the heterotopic implantation of prosthetic valves in the vena cava. These approaches are particularly limited to the patients with degeneration of previous TV correction (repair or replacement) and particularly for those with very advances stages of TR where repair is not going to provide a durable solution (88, 89).

The coaptation and annuloplasty devices represent other available options of the transcatheter TV repair.

**Figure 3:**
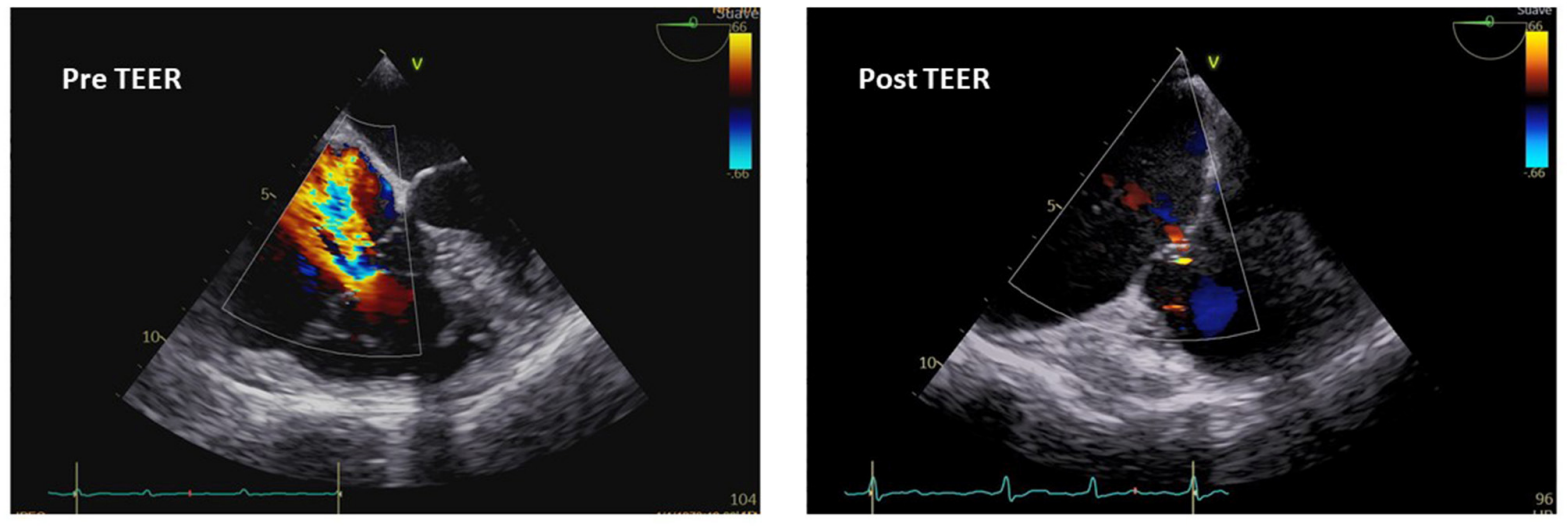
Transesophageal echocardiography imaging and pre- and postoperative assessment of TR after transcatheter edge-to-edge repair (TEER).

The MitraClip system (Abbott Vascular, Santa Clara, California, USA) has found in the TriClip system (Abbott, Chicago, Illinois, USA), the corresponding leaflets coaptation device. In particular, the edge-to-edge repair of the TV with the TriClip system (Abbott, Chicago, Illinois, USA) or the Pascal system (Edwards Lifesciences, Irvine, California, USA) is the most largely performed coaptation technique (Figure 3), with retrospective and prospective analysis that demonstrated a reduction in TR and improvement in the functional class (90, 91). The Triluminate Pilot Study (92) prospectively enrolled symptomatic patients with TR ≥ 2 not suitable for surgery, to evaluate the safety and performance of the TriClip device at 1-year follow-up. The durability of the TriClip system in the follow-up was sustained with the 87% of patients that experienced a TR reduction of ≥ 1 grade, with torrential or massive TR less prone to achieve a satisfactory correction rather than severe TR. Besides TR grade reduction, which remained stable across the follow-up, significant improvements in the clinical status, quality of life, and hospitalization rate have been achieved, further showing reverse RV remodeling (93). Although the results promise well, study limitations such as the small sample size and the exclusion of patients with severe PH or high coaptation gap make necessary large randomized controlled study, which are currently being performed in the currently including phase of the Triluminate Pilot Study where patients are randomized to receive either medical treatment or edge-to-edge repair with the TriClip system.

On a scaled-down, the early feasibility of other TTVR system, conceptualized in order to improve the leaflet coaptation, has been achieved in smaller sample size with the Forma system (Edwards Lifesciences, Irvine, California, USA). The regurgitant orifice area results reduced with a balloon spacer anchored to the RV apex (87, 94). Besides the limited early good results, the invasiveness of the device raised different perplexity mainly in the setting of unsuccessful procedure.

On the other hand, the suture annuloplasty devices, Trialign (Mitralign Incorporation, Tewksbury, Massachusetts, USA) and Tricinch (4Tech Cardio Ltd., Galway, Ireland, UK), provide to counteract TA dilatation, reducing the orifice dimension with a system of anchors placed on the anterior and posterior segments (95, 96).

The release of annuloplasty ring devices was performed by using the Cardioband system (Edwards Lifesciences, Irvine, California, USA) and the Millipede IRIS system (Millipede Incorporation, Santa Rosa, California, USA). At early follow-up, the impact on TR and clinical status was promising in high-risk surgical patients deemed inoperable, including those who underwent prior surgical repair showing residual or recurrent TR.

The analysis of data collected on patients undergoing any type of TTVI (>70% transcatheter edege-to-edge repair) in multiple centers in Europe and North America was performed by Taramasso et al. (83), showing a superiority at 1-year follow-up of transcatheter approach vs. medical management, when procedure results successful. The population study was composed by high-risk surgery patients with FTR in the majority of cases (95.2%). The main outcomes achieved by the study were low mortality, high procedural safety, and significant improvements in the functional class at early- and mid-term follow-up (97–99).

Nevertheless, similar outcomes to those managed medically have been showed in patient with unsuccessful TTVI. Interestingly, a coaptation depth > 1 cm has been identified as predictive of technical failure, suggestive of excessive valve tethering and RV remodeling, expression of the late phase of disease (85). The development of new transcatheter prosthesis will probably address these stages of the disease.

Percutaneous or transthoracic insertion of a bioprosthesis to replace the nonrepairable TV has been attempted in several patients successfully. Sizes up to 54 F required are indeed a limitation to solve in coming years (100).

These findings emphasize the necessity to improve knowledge regarding FTR pathophysiology, in order to better understand the role of TTVI in the natural history of the disease.

## Conclusion

The concept of TV as a passive bystander to the LHVD should be abandoned. FTR is a multifaceted disease, including a broad spectrum of lesions. The technological advances in cardiac imaging with the use of 3D echocardiography and CMR have restored notoriety to the study and management of the FTR and may ensure to accurately categorize FTR phenotypes and establish quantitative parameters useful to guide clinicians and surgeons.

Annuloplasty has demonstrated to be limited to address FTR in its early stage with the necessity to integrate the repair technique by using a more targeted approach, which ensures an improved valvular coaptation counteracting excessive leaflet tethering. Nevertheless, the use of annuloplasty with prosthetic ring may successfully treat TA dilatation in the setting of mild-to-moderate FTR when left-sided surgery is performed.

Therefore, the role of surgery in the transcatheter era, with TTVI representing a valid option for high-surgical risk patients, depends mostly by the correct understanding of the natural history of disease, with a growing armamentarium of techniques in the surgeon's hands.

## Author Contributions

MV and EG: conceptualization. MV, MS, BD-X, and JL: writing—original draft preparation. EG, FM, SR, EW, and MD'A: review and editing. All authors contributed to the article and approved the submitted version.

## Conflict of Interest

The authors declare that the research was conducted in the absence of any commercial or financial relationships that could be construed as a potential conflict of interest.

## Publisher's Note

All claims expressed in this article are solely those of the authors and do not necessarily represent those of their affiliated organizations, or those of the publisher, the editors and the reviewers. Any product that may be evaluated in this article, or claim that may be made by its manufacturer, is not guaranteed or endorsed by the publisher.
